# Deferoxamine Compensates for Decreases in B Cell Counts and Reduces Mortality in Enterovirus 71-Infected Mice

**DOI:** 10.3390/md12074086

**Published:** 2014-07-07

**Authors:** Yajun Yang, Jing Ma, Jinghui Xiu, Lin Bai, Feifei Guan, Li Zhang, Jiangning Liu, Lianfeng Zhang

**Affiliations:** 1 Key Laboratory of Human Diseases Comparative Medicine, Ministry of Health, Institute of Laboratory Animal Science, CAMS & Comparative Medicine Centre, Peking Union Medical College, Beijing 100021, China; E-Mails: yangyajun484@hotmail.com (Y.Y.); alanyingtao519@163.com (J.M.); xiujinghui1984@163.com (J.X.); bailin49@163.com (L.B.); guanff@cnilas.org (F.G.); zhangl@cnilas.org (L.Z.); liujn@cnilas.org (J.L.); 2 State Key Laboratory of Bioactive Substances and Functions of Natural Medicines, Institute of Materia Medica, Peking Union Medical College and Chinese Academy of Medical Sciences, Beijing 100050, China; 3 Department of Medicinal Chemistry, Beijing Key Laboratory of Active Substances Discovery and Drugability Evaluation, Institute of Materia Medica, Peking Union Medical College and Chinese Academy of Medical Sciences, Beijing 100050, China

**Keywords:** deferoxamine, enterovirus 71, hand, foot and mouth disease, B cells

## Abstract

Enterovirus 71 is one of the major causative agents of hand, foot and mouth disease in children under six years of age. No vaccine or antiviral therapy is currently available. In this work, we found that the number of B cells was reduced in enterovirus 71-infected mice. Deferoxamine, a marine microbial natural product, compensated for the decreased levels of B cells caused by enterovirus 71 infection. The neutralizing antibody titer was also improved after deferoxamine treatment. Furthermore, deferoxamine relieved symptoms and reduced mortality and muscle damage caused by enterovirus 71 infection. This work suggested that deferoxamine has the potential for further development as a B cell-immunomodulator against enterovirus 71.

## 1. Introduction

Enterovirus 71 (EV71) is a single-stranded positive-sense RNA virus that belongs to the Picornaviridae family [1]. EV71 is one of the causative agents of exanthema in young children and infants [2], also known as hand, foot, and mouth disease (HFMD). Generally, this disease is mild and self-limiting, but EV71 infections may also lead to various types of neurological complications, such as aseptic meningitis, encephalitis, poliomyelitis-like paralysis, and even death [3]. In recent years, EV71 outbreaks have occurred in the Asia-Pacific region [4]. Unfortunately, no effective vaccines or antiviral drugs are currently available to treat HFMD in the clinic [5].

**Figure 1:**
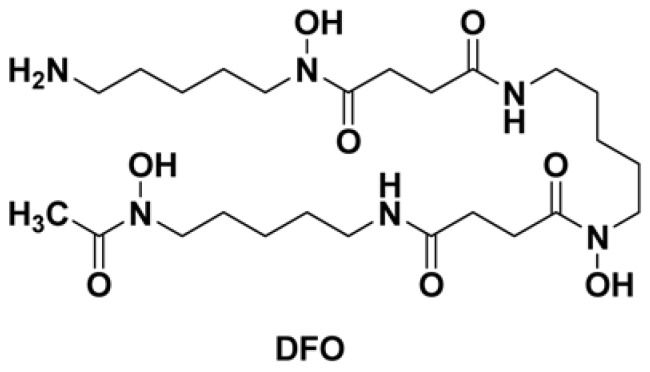
The chemical structure of deferoxamine (DFO).

Infants and young children are highly susceptible to EV71infection. Various factors, especially humoral and cellular immune responses, can affect the pathogenesis of EV71 infection. The significance of lymphocyte and antibody responses in the pathogenesis of EV71 has been determined [6]. Three types of lymphocytes, namely B cells, CD4+ T cells, and CD8+ T cells, can reduce the mortality and tissue viral loads of infected mice [7], suggesting that compounds with immunomodulatory activity might have the potential for further development as antiviral agents against EV71. In this work, we found that deferoxamine (DFO, Figure 1) could compensate for the decreased levels of B cells caused by EV71 infection, which resulted in an increase in the titer of neutralizing antibodies against EV71. Treatment with DFO relieved symptoms and reduced mortality, as well as generated an obvious reduction in EV71-induced muscle damage.

## 2. Results and Discussion

### 2.1. DFO Treatment Reduced Mouse Mortality upon Lethal EV71 Challenge

**Figure 2:**
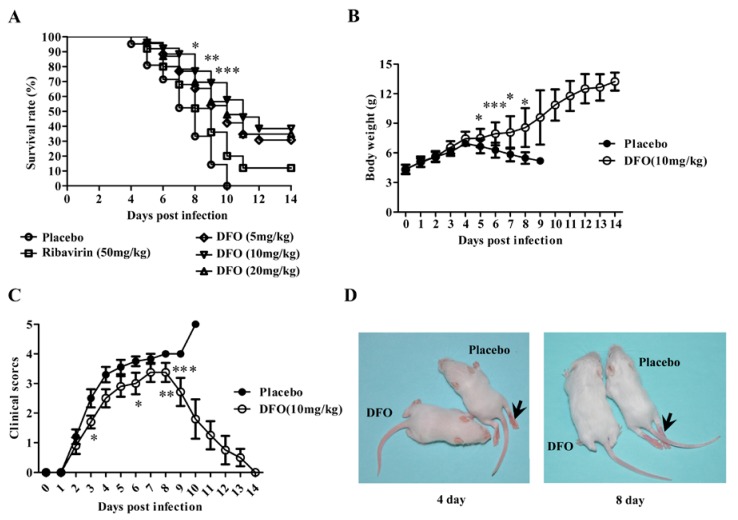
DFO treatment reduced the mortality and relieved the symptoms of EV71-infected mice. (**A**) The survival rates of the EV71-infected mice treated with placebo, ribavirin (50 mg/kg) or DFO (5, 10, or 20 mg/kg) were recorded at 14 dpi (*n* = 80, *: *p* < 0.05, **: *p* < 0.01, ***: *p* < 0.005); (**B**) The body weights of the infected mice treated with the placebo or DFO (10 mg/kg) were recorded in independent experiments (*n* = 20, *: *p* < 0.05, ***: *p* < 0.005); (**C**) the clinical scores of the infected animals were systematically evaluated (*: *p* < 0.05, **: *p* < 0.01, ***: *p* < 0.005); (**D**) The typical phenotype of ruffled hair and paralysis of hind limbs caused by EV71 infection at 4 and 8 dpi (indicated by arrow) is shown, and the symptoms were prevented in the DFO-treated group.

The placebo-treated mice developed paralysis at 3 dpi, and all animals within this group died within 10 dpi. Ribavirin, as a positive control, increased the survival rate of the infected mice to 12%. As shown in Figure 2A, DFO clearly reduced the mortality of the infected mice. When administered at a dose of 5, 10 or 20 mg/kg daily for five consecutive days, the survival rates were 30.8%, 38.5% and 34.8% after two weeks, respectively. The efficacy of DFO was also tested on RD cells infected with EV71. The results indicated that DFO could not inhibit EV71 viral replication *in vitro* (data not shown).

### 2.2. DFO Treatment Relieved the Symptoms of the Infected Mice

The protection of the infected mice that received DFO was further evaluated. In this experiment, each mouse was weighed, and clinical symptoms were scored daily for two weeks. Figure 2B–D illustrate these changes. The placebo-treated mice exhibited higher clinical scores than the mice that received DFO. Similarly, treatment with DFO obviously ameliorated weight loss. Moreover, the surviving mice began to recover after 7 dpi, and no evidence of disease was observed in the surviving mice after two weeks.

### 2.3. DFO Treatment Regulates the B Cell Levels of the Infected Mice

**Figure 3:**
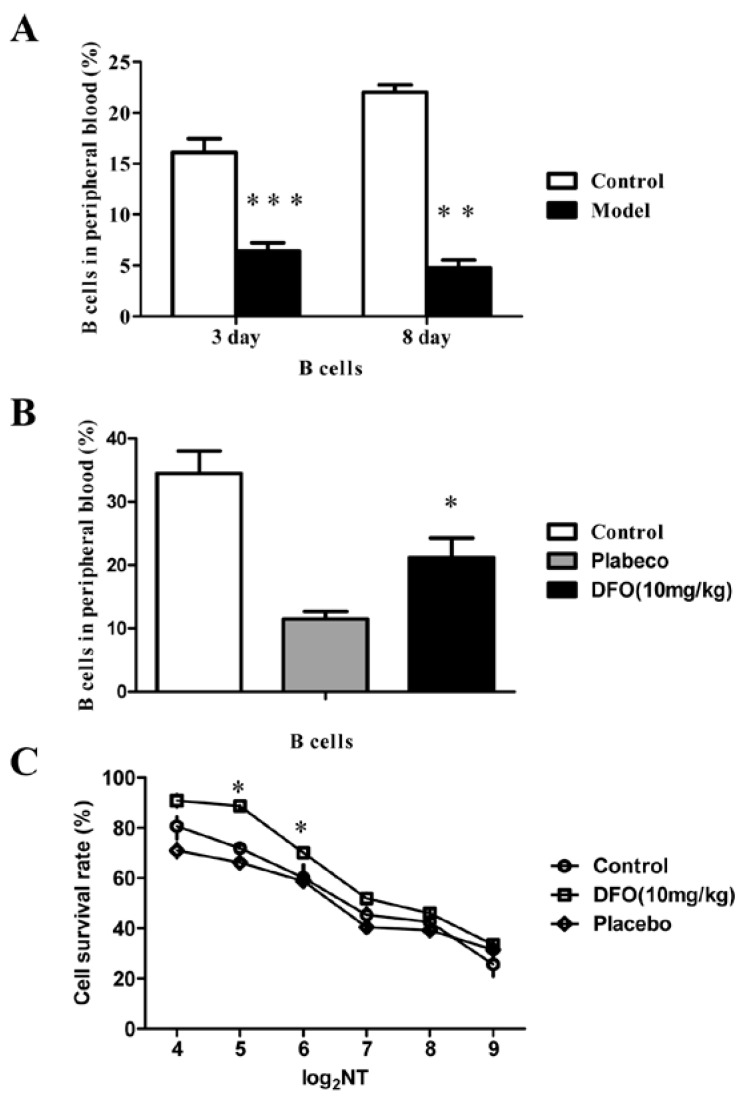
DFO ameliorated the B cell levels in the infected mice. (**A**) The uninfected (Control) and infected (Model) mice were recorded at 3 and 8 dpi, and the levels of B cells were obviously inhibited in human EV71-infected mice (*n* = 20, **: *p* < 0.01, ***: *p* < 0.005); (**B**) The level of B cells was significantly increased after treatment with DFO (*n* = 30, *: *p* < 0.05); (**C**) The neutralizing antibody titer of the DFO group was significantly higher than that of the control and placebo groups (*n* = 30, *: *p* < 0.05).

**Figure 4:**
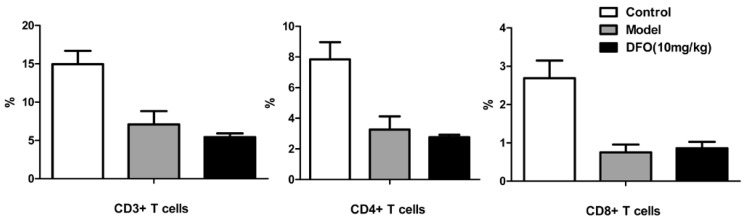
DFO did not alter T cells levels in the infected mice. The uninfected (Control), infected (Model) and DFO-treated mice were recorded at 8 dpi (*n* = 30).

We also investigated the level of B cells in a mouse model of lethal EV71 infection, in which the 10-day-old mice were infected with the mouse-adapted EV71 strain MP10. The levels of B cells from the noninfected mice (Control) and the infected mice (Model) were comparatively analyzed by flow cytometry (Figure 3A), and the results indicated that B cell counts were obviously reduced in EV71-infected mice at three and eight days post infection (dpi). The results suggested that infection with EV71 inhibits B cell counts, at least in mice. To determine whether the therapeutic effect of DFO is related to immunoregulation, we analyzed the changes in lymphocyte numbers in the infected mice (Figure 3B). After DFO treatment, the levels of B cells, which were inhibited by EV71-infection, were significantly increased. Next, a neutralization assay using the sera from mice treated with or without DFO was carried out on human rhabdomyosarcoma cells (RD). The neutralizing titer (NT) of the antibodies against EV71 in the sera was significantly increased, corresponding with the increase in B cells, in the mice of the DFO-treated group, compared with that of the control and placebo groups (Figure 3C). However, the levels of CD3+, CD4+ and CD8+ T cells were not obviously altered after treatment with DFO (Figure 4).

### 2.4. DFO Treatment Slightly Reduced the Viral Load of the Infected Mice

**Figure 5:**
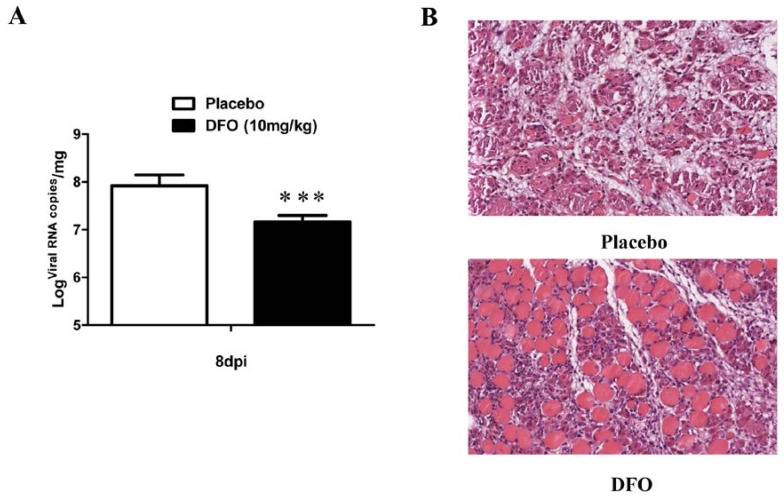
DFO treatment reduced the viral load and muscle damage in the infected mice. (**A**) The infected mice were treated with the placebo or with DFO at a dose of 10 mg/kg, and the muscles were sampled and subjected to viral RNA copy analysis by qRT-PCR at 8 dpi (*n* = 20, ***: *p* < 0.005); (**B**) The pathological changes in the muscle tissues at 8 dpi were observed after H & E staining.

An additional experiment was performed to analyze the viral load and muscle damage in infected mice that received saline or DFO. As shown in Figure 5A, the viral replication in the muscle tissues of DFO-treated mice was slightly inhibited, compared to placebo-treated mice by quantitative polymerase chain reaction with quantitative real-time reverse transcription (qRT-PCR). Obviously, this slight change was not sufficient to relieve the symptoms of the infected mice.

### 2.5. DFO Treatment Reduced the Muscle Damage of the Infected Mice

As shown in Figure 5B, DFO treatment generated an obvious reduction in the muscle damage caused by EV71 infection (8 dpi). In the infected, placebo-treated mice, widespread degeneration, necrosis of muscle cells, severe inflammation and interstitial edema were observed; however, DFO-treated mice only exhibited local degeneration of muscle cells and moderate inflammation.

The role of B cells as antigen-specific effector immune cells in the host defense against pathogens is well recognized. Antibody production by B cells plays a significant role in providing immunoprotection [8]. B cells have been reported to protect mice from EV71 infection. Mice deficient in B cells were highly susceptible to viral infection, and the extent of neutralizing antibodies produced by B cells have also been reported as one of the most important factors protecting against EV71 infection [7]. Antibody therapy has demonstrated favorable effects in animal experiments and clinical practice [9]. In this study, we found that infection with EV71 reduced the levels of B cells in mice, suggesting that the decrease in B cells might enhance the pathological development of HFMD. Therefore, compounds that induce an upregulation in B cell numbers could serve as potential therapeutic agents against EV71.

Marine microorganisms are a major source for marine natural products discovery [10]. As a marine microbial natural product, DFO, derived from *Streptomyces pilosus*, has been approved by the U.S. Food and Drug Administration (FDA) for the treatment of iron overload [11]. In addition to iron chelation, DFO has proven useful for the inhibition of different pathogens, including bacteria, fungi and viruses, due to its immunomodulatory activity in various infected animal models [12]. DFO has convincingly exhibited antiviral and immunomodulatory effects *in vitro* and *in vivo*, and its immunomodulatory activity differs depending on the type of pathogen being treated [12]. Here, we found that DFO compensated for the decreased levels of B cells caused by EV71 infection and improved the levels of the neutralizing antibodies against EV71. The clinical symptoms, muscle damage and mortality, of the infected mice were clearly ameliorated by the DFO treatment. To explore the mechanism of DFO’s antiviral activity, we also analyzed the direct inhibition of EV71 replication in RD cells. DFO could not obviously inhibit viral replication *in vitro*. In contrast, viral replication in the muscle tissues of DFO-treated mice was slightly inhibited. These results suggested that the possible mechanism of DFO activity against EV71 in infected mice was through the upregulation of B cells but not the direct inhibition of EV71.

## 3. Experimental Section

### 3.1. Cells, Viruses and Reagents

Human RD cells were maintained in Dulbecco’s modified Eagle’s medium (DMEM) containing 10% fetal bovine serum (FBS). A clinically isolated EV71 strain FY0805 (GenBank accession No. HQ882182) and the mouse-adapted EV71 strain MP10 (GenBank accession no. HQ712020) derived from FY0805 were cultured in RD cells. The viral titers were determined using a plaque assay as described and working stocks of virus containing 109 TCID50/mL were prepared for experiments. Deferoxamine mesylate (purity > 95%) was purchased from Sigma-Aldrich Inc. (Saint Louis, MO, USA). Ribavirin (purity > 98%) was purchased from the National Institute for the Control of Pharmaceutical and Biological Products (Beijing, China).

### 3.2. Antiviral Assay in RD Cells

For the antiviral assay [13], RD cells (2 × 10^4^ cells/well) were plated in 96-well plates with DMEM medium lacking antibiotics and were grown overnight to 90% confluence at 37 °C. The RD cells were then infected with 100 TCID_50_ of FY0805 and cultured continually in DMEM medium containing 2% FBS. The infected cells were treated with varying concentrations of DFO in saline. The half maximal inhibitory concentration was defined as the concentration of DFO that caused a 50% reduction in the virus’ cytopathic effect, as compared to that of the saline-treated control.

### 3.3. Mouse Protection Assay

Ten-day-old ICR mice were bred in an AAALAC-accredited facility and all of the animal protocols were approved by the Institutional Animal Care and Use Committee of the Institute of Laboratory Animal Science, Peking Union Medical College.

For the lethal EV71 challenge, each 10-day-old mouse was intraperitoneally (i.p.) inoculated with 1 × 10^7^ TCID50 (lethal dose) of MP10. At 2 h postinfection, the infected mice were injected (i.p.) with different concentrations of DFO in saline once daily for six days. The placebo group was injected with the same volume of saline. Ribavirin (50 mg/kg) was used as a positive control. The survival rates of the mice were monitored daily for two weeks.

In additional experiments, the clinical scores were graded as follows: 0, healthy; 1, ruffled hair; 2, weakness in hind limbs; 3, paralysis in a single hind limb; 4, paralysis in both hind limbs; and 5, death [14]. After euthanasia, the muscle tissues of the mice were sampled for virology and pathology analysis, and the blood was analyzed by flow cytometry.

### 3.4. Flow Cytometry

The cells from peripheral blood were filtered through a sterile nylon mesh, and stained with the indicated antibodies. Data acquisition was performed on a FACS Aria I (Becton Dickson) and analyzed using the FlowJo software. The antibodies used for staining were FITC B220 (RA3-6B2), PerCP-Cy5.5 CD3e (145-2C11), FITC CD8 (53-6.7), PerCP-Cy5.5 CD4 (RM4-5). All antibodies were obtained from eBioscience.

### 3.5. Neutralization Assay

Mouse sera were collected at 8 dpi to determine neutralizing titers as previously described [15]. Briefly, serial two-fold dilutions of heat-inactivated serum were mixed with a two-fold volume of a 100% infectious dose of EV71. The serum and virus mixtures were incubated at 37 °C for 1 h before 1 × 10^4^ RD cells were added. The cultures were incubated for 48 hours and stained with crystal violet to observe cytopathic effect.

### 3.6. Determination of the Viral Load

qRT-PCR was used to detect the viral RNA copy number [16]. Briefly, the total RNA was isolated from the muscle tissues of the mice using the TRIzol reagent (Sigma-Aldrich, Saint Louis, MO, USA). The total RNA was then reverse transcribed using random hexamers with a reverse-transcription kit (Promega, Madison, WI, USA). The cDNA was subjected to quantitative PCR (QuantiTect SYBR Green RT-PCR kit, QIAGEN, Duesseldorf, Germany) with a Roche LightCycler 3.5 system for 40 cycles. The primers were EV71-S1 (5′-AGATAGGGTGGCAGATGTAATTGAAAG-3′) and EV71-A1 (5′-TAGCATTTGATGATGCTCCAATTTCAG-3′). A fragment corresponding to nucleotides 2462–2635 of FY0805 was used as a standard curve (1 × 10^1^ copies/μL to 1 × 10^8^ copies/μL) and was used as a standard to calculate the viral RNA copy number.

### 3.7. Pathology

After euthanasia, the muscle tissues of the mice were immediately immersion-fixed in 10% buffered formalin for 48 h. The tissues were bisected, embedded in paraffin, and stained with hematoxylin and eosin stain (H & E). Ten sections of muscle were observed per animal in a blinded manner.

### 3.8. Statistics

All data are expressed as the mean ± S.D. The statistical significance of differences in the mean values was assessed by Duncan’s multiple range tests, following a one-way analysis of variance (ANOVA), and survival rates were analyzed by GraphPad Prism 5 (GraphPad Software, La Jolla, CA, USA). A *p*-value of <0.05 was considered to be significant.

## 4. Conclusions

Many strategies have been developed recently to address the EV71 epidemic; however, no effective therapy procedures have been clinically available as yet. Considering cost and safety, the discovery of effective therapeutic agents from existing drugs is an acceptable strategy. The current study demonstrated that DFO might be a promising candidate for development as an immunomodulator to treat EV71 infection.
